# Synaptic facilitation and learning of multiplexed neural signals

**DOI:** 10.3389/fnetp.2025.1664280

**Published:** 2025-10-23

**Authors:** Nigel Crook, Alexander D. Rast, Eleni Elia, Mario Antoine Aoun

**Affiliations:** ^1^ Institute for Artificial Intelligence, Data Analysis and Systems (AIDAS), School of Engineering, Computing and Mathematics, Oxford Brookes University, Oxford, United Kingdom; ^2^ Independent Researcher, Montreal, QC, Canada

**Keywords:** temporal coding, spiking networks, synaptic plasticity, channel capacity, interspike intervals, neural coding, rate coding, network physiology

## Abstract

**Introduction:**

In this work, we introduce a novel approach to one of the historically fundamental questions in neural networks: how to encode information? More particularly, we look at temporal coding in spiking networks, where the timing of a spike as opposed to the frequency, determines the information content. In contrast to previous temporal-coding schemes, which rely on the statistical properties of populations of neurons and connections, we employ a novel synaptic plasticity mechanism that allows the timing to be learnt at the single-synapse level.

**Methods:**

Using a formal basis from information theory, we show how a phase-coded spike train (relative to some ‘reference’ phase) can, in fact, multiplex multiple different information signals onto the same spike train, significantly improving overall information capacity. We furthermore derive limits on the channel capacity in the phase-coded spiking case, and show that the learning rule also has a continuous derivative in the input-output relation, making it potentially amenable to classical learning rules from artificial neural networks such as backpropagation.

**Results:**

Using a simple demonstration network, we show the multiplexing of different signals onto the same connection, and demonstrate that different synapses indeed can adapt using this learning rule, to specialise to different interspike intervals (i.e., phase relationships). The overall approach allows for denser encoding, and thus energy efficiency, in neural networks for complex tasks, allowing smaller and more compact networks to achieve combinations of tasks which traditionally would have required high-dimensional embeddings.

**Discussion:**

Although carried out as a study in computational spiking neural networks, the results may have insights for functional neuroscience, and suggest links to mechanisms that have been shown from neuroscientific studies to support temporal coding. To the best of our knowledge, this is the first study to solve one of the outstanding problems in spiking neural networks: to demonstrate that distinct temporal codings can be distinguished through synaptic learning.

## 1 Introduction

### 1.1 The problem of learning

#### 1.1.1 Why spiking?

‘Neural networks’ encompass a wide variety of techniques involving massively-parallel computing models and, crucially, learning. Despite decades of impressive progress, however, efficient learning remains elusive. Although in the ‘classical’ domain of perceptron-style neural networks backpropagation has provided an effective general learning algorithm, the fact remains that modern models require numerous epochs of training involving billions of weight modifications introduced through the presentation of thousands to millions (or more) of data examples. It would be a stretch to call such learning schedules ‘efficient’, although they may be effective, i.e., produce high-accuracy output results. Biological neural networks, the supposed prototype of the genre, clearly do not require such large-scale data presentations and are achieving comparable results at far greater efficiency, so clearly something very different is going on in the biology. Whilst large-scale models based on backpropagation running on very large machines may be reasonable for offline inference-based responses to query-type tasks, edge computing, on small, embedded devices, clearly needs something different. A closer look at the biology is worth it here to find more efficient, smaller-scale neural solutions to real-time, real-world problems. But what, exactly, is biology doing?

A growing body of research and practice emphasises the fundamental difference in information representation: spiking, in contrast to continuous signals, as a crucial advantage of biology (Tang et al., 2017). It has been repeatedly suggested that two critical properties of a spiking representation, namely, its event-driven nature, and its higher potential information capacity, open the door to potentially advantageous computational efficiency (Wang and Cruz, 2024; Rathi et al., 2023). As early as 2004, Maass and Markram (2004) showed that by abandoning the discrete-time assumption of classical neural networks, where all computations are performed on equilibrium states, a spiking network can achieve universal computational capability as long as 2 distinct inputs can be discriminated. Despite these theoretical suggestions, however, real progress relative to classical nonspiking models remains elusive. Establishing that inputs are in fact, distinguishable has not proven easy. It has been shown that spiking models can work, but evidence that they can be more efficient than backpropagation-style machine learning is limited (Davidson and Furber, 2021; De Florio et al., 2023). There is thus a strong need for fundamental models of learning in spiking networks that are both efficient and scalable - and compare favourably with classical neural networks.

#### 1.1.2 Why on the synapses?

Most models of learning in neural networks presume that the atomic unit of learning itself is the synapse (in classical neural networks they are frequently termed a parameter), which has historically been approximated as a weighted connection from one neuron to another. A presynaptic neuron transmits some (coded) value to a postsynaptic neuron, multiplied by a (scalar) weight which represents the operation carried out in the synapse. The most common choice, then, to implement learning is to modulate the weight in some way - hence the choice of synapses as the learning element. Although it would be possible to implement learning by a modulation of some other element (e.g., adaptive threshold), changing the synaptic efficacy is usually chosen in spiking networks because it can be linked to the well-researched STDP mechanism and also corresponds well to weight updates in artificial neural networks (ANNs). However, modulating the weight is easy to do if the value being transmitted itself is a scalar, but necessitates a choice if spiking is to be used, since a (single) spike does not naturally map to a scalar representation.

#### 1.1.3 Why temporal?

Spikes generally do not have an amplitude, and thus the possible representations fall into 2 types: rate coding and temporal coding, which are basically the event-driven equivalent of frequency modulation and phase modulation. Rate coding, whilst popular because it is easy to transform an equivalent classical model to a rate-coded model, loses significant efficiency when there is nonzero phase noise, because then multiple spikes have to be received in order to define a mean rate (Maass and Orponen, 1998). Symbol-to-symbol transitions also require some ‘dead’ space whilst the rate is transient, thus further limiting the rate of information transmission and hence efficiency. Temporal coding, by contrast, can achieve efficiency limited only by the phase noise in the network, and thus would seem like the more promising direction where efficiency is the goal. However, again, models of temporal learning remain thin on the ground, not least because while in the rate-coding case there is a natural scalar intepretation of the value of a given signal, in the temporal coding case the interpretation is arbitrary. Formally, it is possible to encode multiple symbols using simple absolute timing (‘Time to Spike’) (Van Rullen et al., 1998), but since in general this would require arbitrary time-to-decode (each symbol might occur at any future time), the interpretation (and number of bits per spike) remains dependent on some choice of maximum delay. It therefore makes sense to define a model for temporal learning that on the one hand can work with individual spikes, and on the other, is not dependent on any particular choice of representation of the temporal code. This can be done by transforming the problem of learning from one of adjusting weights - the synaptic transmission strength - to one of adjusting delays - the synaptic transmission timing. With the goal being to create a model for temporal learning, strict biological realism or close adherence to biological plausibility can be relaxed in favour of creating models that demonstrate the possibility, in principle, of temporal learning, which might subsequently be used to inform either neurobiological studies or computing applications. This paper introduces a simple synaptic model for this purpose, and shows how such models can be used to learn temporally sensitive neural decoding mechanisms.

### 1.2 Background

#### 1.2.1 Neural coding schemes: rate coding vs. temporal coding

Neural coding describes how neurons encode and transmit information, with two primary schemes being rate coding and temporal coding. Rate coding relies on the average firing rate of neurons over a specific time window, where the frequency of spikes represents the encoded information (Gautrais and Thorpe, 1998). This approach is robust and simple, but limited in rapid sensory processing due to its reliance on longer observation periods (Van Rullen and Thorpe, 2001). In contrast, temporal coding emphasises the precise timing of spikes, which can carry significantly more information, particularly in rapid sensory processing and working memory mechanisms, and is suitable for spiking neural models and neuromorphic systems (Guo et al., 2021). There is significant evidence from biology that temporal coding is important in processing, and leads to a dramatic increase in information capacity (Kayser et al., 2009). The authors showed not only higher information capacity, but also that the resultant coding was more robust to noise, making a compelling case for using phase coding in the presence of noisy inputs. Surprisingly, however, such schemes have been but little used in computing systems or machine intelligence, where there is the potential not only to improve data rates at inference time (i.e., after learning) and robustness during training (i.e., before learning), but also potentially to inform further work in computational neuroscience. Much of the reason for this apparent lack of uptake may be the challenges involved in identifying suitable learning rules.

#### 1.2.2 Temporal coding in computational neural models

Various computational models based on temporal coding have been investigated. As early as 1993, Judd and Aihara (1993) introduced the so-called pulsed propagation network (PPN). Unlike neural network models that rely on average firing rates, the PPN uses time intervals between action potentials as continuous values. The authors showed that PPNs are computationally more powerful than Turing machines and capable of approximating so-called ‘R-machines’, which are general computing machines that apply algorithms to data consisting of real-valued numbers.

There has always been a strand of models that are pure machine translations of biological systems. For example, Rabang and Bartlett (2011) developed a computational model of thalamocortical neurons in the medial geniculate body (MGB) which transforms temporal coding from synchronized inputs in the inferior colliculus (IC) to rate-coded outputs in the MGB. The authors report that large-conductance IC inputs preserve synchrony while small-conductance inputs desynchronize and filter temporal modulation. By varying synaptic properties, input jitter, and membrane potential, they highlight how distinct cellular mechanisms shape auditory temporal processing in the MGB.

Other models aim at replicating important computational processes. One of the most well-studied classes of temporal spiking networks are the ‘projection’-style networks of which reservoir computers (Jaeger et al., 2007; Maass et al., 2002) and polychronization (Izhikevich, 2006) are the most well-known. These relied on synaptic adaptation to tuned delays generated in a random network; in the polychronization case by path selectivity, in the reservoir case by output selectivity. The physical delays themselves, however were fixed for any given path (generally initialised randomly) and did not change over the course of processing; such models worked by creating a sparse projection into a high-dimensional space and thus the information capacity of the network was typically limited by the number of connections. On the one hand, such models are easily translated into relatively realistic and plausible biological networks because the (fixed) delays can be modelled as different axon lengths or spine locations along the dendrite. On the other hand, however, the information storage capacity of these models is typically a small fraction of the number of connections, and accuracy as well as capacity is limited by the delay combinations that happen to exist in the instantiated network. Therefore, such models are very useful in demonstrating the possibility of temporal coding and learning but are not particularly efficient (typically demonstrating lower information capacity per connection than deep networks, even though the latter exploit similar high-level theoretical properties (Penkovsky et al., 2019)).

Millidge et al. (2024) introduced a computational model called temporal predictive coding (tPC) that extended predictive coding to process dynamically changing sensory inputs over time. In a predictive application, the tPC model achieved performance comparable to a Kalman filter and demonstrated an ability to learn motion-sensitive, Gabor-like receptive fields from natural dynamic inputs. In Comşa et al. (2021), the authors introduced spiking autoencoders that utilized temporal coding to process and reconstruct images with high fidelity, leveraging the relative timing of neuronal spikes for information encoding. They used a biologically-inspired synaptic transfer function with backpropagation, achieving performance comparable to conventional artificial neural networks on MNIST and FMNIST datasets. The same group earlier presented a computational model based on a spiking neural network that encodes information in the relative timing of individual spikes (Comşa et al., 2020), using a biologically-inspired alpha synaptic transfer function and trainable synchronization pulses for temporal references. Such networks illustrate the potential for spiking networks using temporal coding to solving complex tasks. Comşa et al. (2020) accomplish the MNIST digit classification task using a variety of coding strategies, encoding pixel brightness values as temporal delays, and digit classifications based on the timing of the first output neuron to spike. However, in spite of promising performance, such demonstrations remain relatively small scale, particularly compared to the advances made in network size and application capability using conventional multilayer perceptron-style networks trained using backpropagation.

#### 1.2.3 Artificial neural networks vs. spiking neural networks

The currently dominant method in machine intelligence, namely, Artificial Neural Networks (ANNs), relies on synaptic weights that are synchronously updated in discrete time in order to set the connection strength between neurons, which determine the processing and learning of information. Optimization of weight modification strategies in ANNs has been shown to improve performance and adaptability. Rumelhart et al. (1986), building on the seminal work of Hebb (1949), advanced the understanding of ANN learning mechanisms through the popularisation of backpropagation (earlier introduced in Bryson and Ho (1969); Werbos, 1974). Backpropagation allows ANNs to adjust synaptic weights efficiently (indeed, optimally) improving generalisation and learning (although notoriously susceptible to the problems of overfitting and ‘vanishing gradients’). Whereas ANNs transmit continuous-valued signals, in Spiking Neural Networks (SNNs) transmission of information happens through discrete spikes. This reduces energy consumption and makes them potentially more computationally efficient Maass (1997). Where in an ANN, a weight (or ‘parameter’) is typically a deterministic multiplier of the input signal, in SNNs the comparable rôle of synapses (and hence weights) is to modulate the probability of spike transmission. This, in turn, influences the dynamics of the network and the learning capabilities, enabling efficient processing of information through relatively sparse (compared to classical ANNs) but informative synaptic connections.

#### 1.2.4 Hebbian learning, long term potentiation and spike‐timing‐dependent‐plasticity

Donald Hebb postulated, in his book “The organization of behavior”, in 1949, that when neuron cells, A and B, keep mutually exciting each other, then a bond is formed between both cells such that whenever A fires, B fires, and *vice versa* (Hebb, 2005). Subsequently, Bliss and Lømo (1973) demonstrated Long Term Potentiation (LTP) which describes the increase in synaptic strength between neurons after prolonged stimulation of their synapses. Later, towards the new millennium, Markram and Sakmann (1995), Markram et al. (1997) and others (Gerstner et al., 1996; Bi and Po, 1998) hypothesised that the exact timings of excitation or inhibition between neurons played an essential role in synaptic modifications, introducing the mechanism generally known as spike-timing-dependent plasticity (STDP) (Markram et al., 2012), which is the biological (and subsequently, technological) implementation of Hebbian learning. As a mechanism, STDP itself is considered to function independently of other synapses, but in the context of the entire network there is biological evidence that in fact, synapses are coupled in ‘clusters’ (Agnes and Vogels, 2024).

#### 1.2.5 Existence of functional synaptic clusters

Functional synaptic clusters are formed through activity-dependent mechanisms, where synaptic inputs with correlated activity are preferentially stabilized and spatially organized into groups, hence clusters (Hedrick et al., 2022). This behavior may enhance the efficiency of information processing (Kastellakis and Poirazi, 2019). During learning, new synapses form near preexisting task-related spines, creating locally coherent activity patterns that encode learned behaviors (Kastellakis et al., 2015). Overall, synaptic clusters appear to amplify presynaptic activity, enabling precise control over postsynaptic responses. However, the majority of existing work has focussed on synchronous synaptic activation, implying an integrator function. Numerous studies consider the population dynamics, as opposed to the processing of individual neurons (Bazhenov et al., 2008), and frequently use a fixed-connectivity model in order to concentrate on the effects of signalling parameters (Brunel, 2000). This may be useful for densely-connected networks with relatively weak couplings operating in a synchronous (essentially, discrete-time) regime (Penn et al., 2016), but discards phase information that may be useful in temporal coding. If, however, such clusters could be used not as integrators but as phase discriminators, sensitive to a particular timing pattern, it becomes possible to implement suitable learning mechanisms for temporally-coded networks.

### 1.3 Biological inspiration

There is considerable evidence that synapses in biological neural networks use mechanisms at multiple time scales, at least some of which introduce timing-sensitive processing capability (Bi and Po, 1998). This contrasts sharply with the majority of computational neural network implementations, whether spiking or non-spiking (continuous-valued), which hitherto have typically treated synapses as (possibly dynamic) amplifiers, but not phase discriminators. That is to say, the transmission characteristics of the synapse do not depend upon the incoming timing of signals. Biological synapses, however, have at least 4 separate potential channels - AMPA (fast [ ∼ 1–10 m] excitatory), GABA-A ([ ∼ 10 m] fast inhibitory), NMDA (slow [ ∼ 100 m] excitatory) and GABA-B ([ ∼ 200 m] slow inhibitory) (Destexhe et al., 1995; Nicoll and Malenka, 1995). The interaction of these different channels has been shown in biological neuroscientific studies to allow for both phase- and frequency-sensitive discrimination of signalling and may be fundamental to sequence learning and timing-sensitive decoding (Romo et al., 1998). Lin and Faber (2002) provide an early indication that changes in synaptic delay may be a factor in (short-term) plasticity within biological networks.

There are plausible mechanisms in biology that could allow a network to learn specific delay patterns, e.g., through preferential strengthening of particular connections dependent upon spine location and dendritic position. Such mechanisms could be incorporated into reservoir-like or polychronisation architectures to learn delay codings. However, such an approach is relatively costly in number of connections per delay encoded, and furthermore what delays can be learnt is critically dependent upon the (built-in) statistics of the delay distributions within the network, because these mechanisms all rely on the ‘right’ delay value happening to exist in the network through some combination of delays extant in the initial system. A more efficient alternative approach would be to encode via synapses themselves becoming tuned to a particular delay, and there are also plausible biological methods to achieve this.

Detailed biophysical models have been introduced (Mäki-Marttunen et al., 2020) that may offer a variety of low-level mechanisms to encode delay, however, the intent in this work being to produce a simple model amenable to computational experimentation across a range of scales, such a level of biological realism is out of scope. Simpler mechanisms, however, can be elaborated which retain some biological plausibility. NMDA channels, in particular, are thought to play a critical rôle in the modulation of spike-timing-dependent plasticity (STDP) (Shouval et al., 2002). The ability of synapses to strengthen or weaken over time, hence their plasticity, is mediated through NMDA-type glutamate receptors (NMDARs) (Malenka and Nicoll, 1999). Biologically, the timing and amplitude of calcium influx through NMDARs play a crucial rule in inducing LTP or Long Term Depression (LTD.) (Kennedy, 2016). The interaction of NMDA thus may affect the time distribution of synaptic transmission, and hence offers a potential mechanism for learning mechanisms that tune the synapse for a particular phase sensitivity (as opposed simply to changing the ‘weight’, considered as the integrated charge release over the entire open channel time). Boudkkazi et al. (2007) observed release-amplitude-dependent variation of synaptic latencies. Their study verified that such variation is induced during plasticity, and determine that the most plausible celluar mechanism is modulation of Ca^2+^ channels. These ideas were initially explored in Crook et al. (2023) by devising a plausible mathematical model loosely based on NMDA/AMPA kinetics. In this work we seek to extend this approach to networks of spiking neurons that learn and are able to decode particular spike sequences, which may overlap on the same axon.

## 2 Materials and methods

### 2.1 Motivation

#### 2.1.1 Theory

**FIGURE 1:**
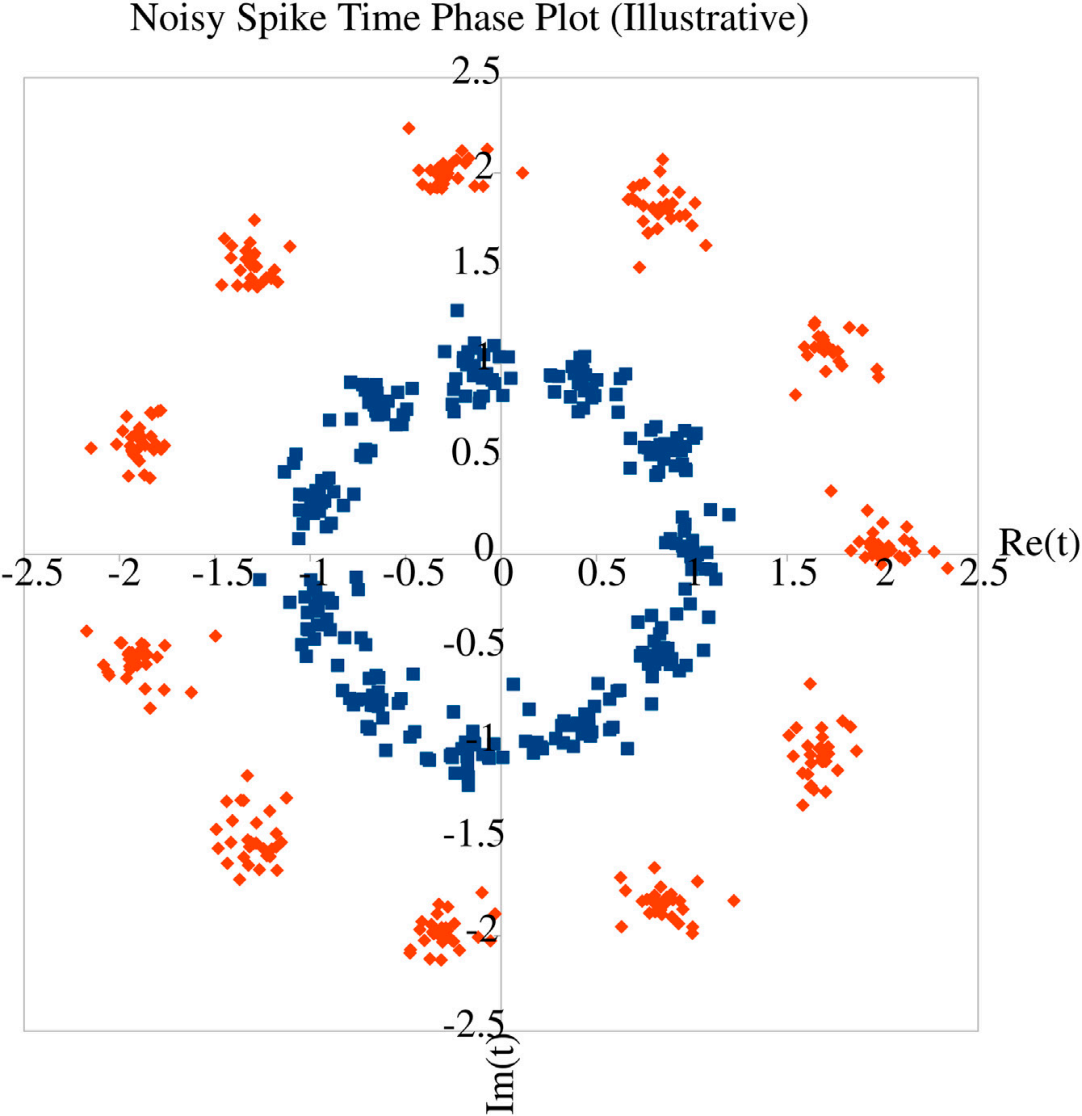
Illustrative figure to demonstrate the insight of Ikeda and Manton (2009). Consider spikes with noisy phase margin relative to some reference timing (often, another spike, but could be, e.g., background oscillations of network activity). In the inner ‘blue’ ring, the phase noise is just below the point where individual phases can be relatively reliably discriminated. With the same temporal noise but a slower reference (outer ‘red’ ring), the discrimination is better, at the cost of reduced information capacity, since the same number of symbols can be decoded at a slower overall rate. Hence higher phase noise entropy increases information capacity. However, the number of distinct phases remains discrete - attempting to detect continuous phases would end up (particularly in the ‘blue’ ring) conflating symbols, hence producing an unreliable decode.

Given the evidence from biology that phase coding increases information capacity (Kayser et al., 2009), the question arises of how much information can be contained in a given spike train. Mijatovic et al. (2021) consider interactions between multiple neurons and find that a continuous-time representation is more accurate and expressive. Nevertheless, Ikeda and Manton (2009) analyse both temporal and rate-coded neurons and find that under some assumptions about the phase noise distributions, the capacities of both are maximised by a discrete input distribution - essentially equivalent to a fixed symbol vocabulary (Figure 1). In the temporal coding case, this distribution is discrete in the complex plane, corresponding to n-ary phase-shift keying modulation where n is set by the underlying phase noise distribution. A classical formula based on information theory for the output of a spiking neuron is given in Equation 1 (Li et al., 2023).

$$H^{T}=-\frac{1}{T}\underset{i}{\sum }P\left(w_{i}\right)log_{2}\left(P\left(w_{i}\right)\right)$$ (1)

assuming the spike train is binned into intervals of time δt within an overall window of time T which defines a ‘word’ $w_{i}$ in the input sequence of length $\frac{T}{\delta t}$ , where $w_{i}$ is a specific binary bit-pattern assumed to have a ‘1’ in the n th position in the window T if a spike occurs with the interval n>t % T≥n−1 (where % is the modulus operator) Such a computation assumes a serial bitstream representation, where a spike’s timing is only relevant insofar as it aligns with a particular bit-slot n . This discards the possibility of a phase-coded representation, where the timing of the spike signals a complete word, and it is further evident that the equation above is reliant on a more-or-less arbitrary choice of the bin interval δt . The study of Ikeda and Manton (2009) is valid in the condition of perfect decoding, but the question arises of information transmission over an unreliable channel, where the probability of decoding a word may be less than 1.

Consider therefore a spike train encoded according to a phase-modulated encoding, where the relevant phase of the spike in a similar window T encodes a complete word. The bit-length of a word that can be successfully decoded within such a window is dependent upon the phase noise or ‘jitter’ $\nu_{\phi }$ of a spike with notional phase ϕ . Consider each spike at time t % T=ϕ to have a Gaussian phase noise distribution N(ϕ) with mean μ=ϕ and variance $\sigma =\nu_{\phi }^{2}$ . In this case, the probability of decoding a given word $w_{n}$ will be found by multiplying the spike train by a sliding Gaussian kernel equivalent to the phase noise distribution - i.e., by a convolution:

$$P\left(w_{n}\right)=S_{n}\left(t\right)\;*N\left(\phi \right)$$ (2)

where $P\left(w_{n}\right)$ is a continuous probability distribution in the word space $W=\left\{w_{n}\right\}$ and $S_{n}\left(t\right)$ is the subpattern of the complete spike train associated with word $w_{n}$ . Thus the capacity of the signal is:

$$H^{T}=-\frac{1}{T}\int_{t}^{t+T}S\left(t\right)\;*N\left(\phi \right)log_{2}\left(S\left(t\right)\;*N\left(\phi \right)\right)dt$$ (3)

where S(t) is the complete spike train between t and t+T . The probability of successfully decoding a given word n with notional phase $\phi_{n}$ is then

$$P_{D}\left(w_{n}\right)=\int_{-\infty }^{\infty }P\left(w_{n}\right)P\left(W\right)dn$$ (4)

where P(W)=S(t) ∗N(ϕ) and is the (prior) probability of any word being decoded, and $P\left(w_{n}\right)$ is the likelihood of word n .

This result is independent of any particular choice of representation W and of any particular assumptions about timing. There is an important choice, however, in the shape of the Gaussian phase noise N(ϕ) . The entropy of a Gaussian is dependent on the variance: $H\left(N\left(\phi \right)\right)=\frac{1}{2}\left(1+log\left(2\pi \sigma \right)\right)$ . The information content of a spiking signal (Equation 3) is dependent on this phase noise convolution kernel - a higher value of σ implies greater potential information content. But this implies in turn a more ‘flattened’ Gaussian which will render the probability density of any given word $w_{n}$ less. Thus there is a tradeoff between how much information can be transmitted (essentially, how many distinct words $w_{n}$ can be recovered) and how probable it is that any given word $w_{n}$ will be decoded successfully. This allows us to build networks with both varying information capacity and varying precision, for different application scenarios.

If an ideal word is defined by a reference spike train $S_{r}$ with message probability distribution $P_{r}\left(W\right)=S_{r}\left(t\right)\;*N\left(\phi \right)$ , we may wish to compute the similarity between this reference word and some actual received spike train, to serve as a basis for learning. Lyttle and Fellous (2011) introduces a similarity measure with strong affinity to Equation 4.

$$d_{SC}=1-\frac{\int_{0}^{L}f\left(t\right)g\left(t\right)dt}{\sqrt{\int_{0}^{L}f{\left(t\right)}^{2}dt}\sqrt{\int_{0}^{L}g{\left(t\right)}^{2}dt}}$$ (5)

where f(t) and g(t) are spike trains convolved with some Gaussian kernel in a similar manner to Equation 2 above. Equation 4 shows that this simply represents the loss in posterior probability that the second spike train f(t) received will be accurately decoded against the first (reference) spike train g(t) .

Decoding a word is equivalent to finding a structure that maximises the probability that the word in question will be identified given a particular spike train. It can be seen from Equation 4 that this will be achieved when $P\left(w_{n}\right)$ is a delta function on n , in other words, when the decoder modulates the input spike train with a phase-shifted Gaussian filter that exactly matches the phase noise distribution N(ϕ) . Indeed, Smirnov et al. (2024) analyse the effect of different synaptic kinetics (and thus sensitivities to phase noise) and find not only that this affects the dynamics, but also, as suggested earlier, that increasingly broader kinetics lead to more complex phase spaces and potentially higher information content. If, therefore, we can create a synapse that can adapt itself such that its transmission probability matches this phase noise distribution with the appropriate phase shift, we can create a functional implementation of a decoder for a given word. If multiple synapses afferent on a given neuron have individually tunable (i.e., learnable) transmission probabilities, tuned to different word-phases, we can create a neuron that can decode an arbitrary set of overlapping words in the input spike train.

We are now in a position to define more formally the difference in spike trains, considered from a point of view of information theory. Given that both convolved functions $P_{r}\left(W\right)=g\left(t\right)$ and P(W)=f(t) represent the word probability distributions within the message, the Kullback-Liebler (KL) divergence gives us a measure of the similarity in the distributions - i.e., how much information difference there is between the two. This is shown in Equation 6:

$$D_{SC}=KL(P_{r}\left(W\right)\|P\left(W\right)=\int_{-\infty }^{\infty }P_{r}\left(W\right)log_{2}\left(P_{r}\left(W\right)/P\left(W\right)\right)dn$$ (6)

This expression for the difference is spike trains has the useful property that it can be used as a loss function for supervised learning approaches, used at the output neurons to compare the output against a canonical ‘reference’ spike train. Furthermore, the recasting of the output into the decode probability domain changes the meaning of the ‘activation’ of a neuron from a non-differentiable discrete event (the neuron either spikes or does not) to a differentiable continuous distribution representing the probability that the neuron will output a given word $w_{n}$ (equivalently, spike at phase ϕ ) for a given input (Equation 4) - enabling efficient backpropagation-style algorithms with spiking neural networks.

#### 2.1.2 Time structuring in spike trains

We now wish to consider a spike train that contains multiple overlapping encoded symbols. It is immediately evident that a rate-coded spike train cannot do so, because rate is not defined over an instantaneous interval, and hence symbols are temporally separated (with a change in spike frequency corresponding to symbol change). Multiple symbols can only (co-)exist if the symbols are encoded by phase: the relative timing of a spike compared to some (possibly implicit) reference timing. A reference timing can easily be generated in spiking networks, e.g., by a neural oscillator or similar mechanism, although we do not concern ourselves here with the details. Each discrete phase ϕ can be discriminated as a different symbol $w_{n}$ , or equally a fixed set of phases $\phi_{0},\phi_{1},\ldots \phi_{m}$ could be interpreted as a single symbol. As long as there is at least one distinct phase in each symbol not shared by other symbols, we may then encode multiple such symbols in the same time period - and multiple messages (groups of symbols) which represent different distinct timing patterns. We refer to these messages as time structured spike trains. The reference timing T will be called a window - i.e., a fixed period within which the time structured spike train is decoded.

One use of such time-structured spike trains is obvious: to encode information with explicit temporal content (such as, e.g., a set of spoken words). However, it is useful to consider a different application of these patterns, when the same input carries multiple features that may be decoded independently. For example, an image could contain separate channels for shape and colour. Although there is no temporal relation as such in the data, using a temporal encoding would allow both sources of information to be carried over the same connections to separate decoding ‘heads’. Effectively, the information would be multiplexed onto the same axon (or in general, downstream connection) and be carried to all subsequent neurons it connects to. Without making any specific conjecture about precisely where (or indeed if) such multiplexing would occur in a biological network, temporal coding allows computational modelling of processes that may be employed in biology as well as computation to make efficient use of limited resource. Not only does this mean fewer neurons and synapses to process the same information stream, it also means that the streams can be treated either separately or jointly, without having to make a prior decision about what classes of information would be useful to downstream processing elements. This capability allows a network using time structured spike trains to retain latent information that may be learned online later. Such a capability stands in sharp contrast to ‘traditional’ neural networks, either spiking or non-spiking, that ‘bake in’ the decoding upstream so that what is even learned in downstream layers is a strict function of the information extracted in the previous layers. Methods such as ‘skip connections’ (He et al., 2016) or recurrent networks (Hochreiter and Schmidhuber, 1997) are often used to try to circumvent this limitation of classical networks, often at the expense of greater computational complexity or stability. In a spiking model, information can be multiplexed at the soma (neuron level) through input from different synapses tuned to different delays, whilst being decodable downstream by separate outputs themselves tuned to the corresponding input delays. A time structured spike train offers an effective way to avoid having to introduce complex connectivity patterns in order to extract features that may not be apparent at the outset.

**FIGURE 2:**
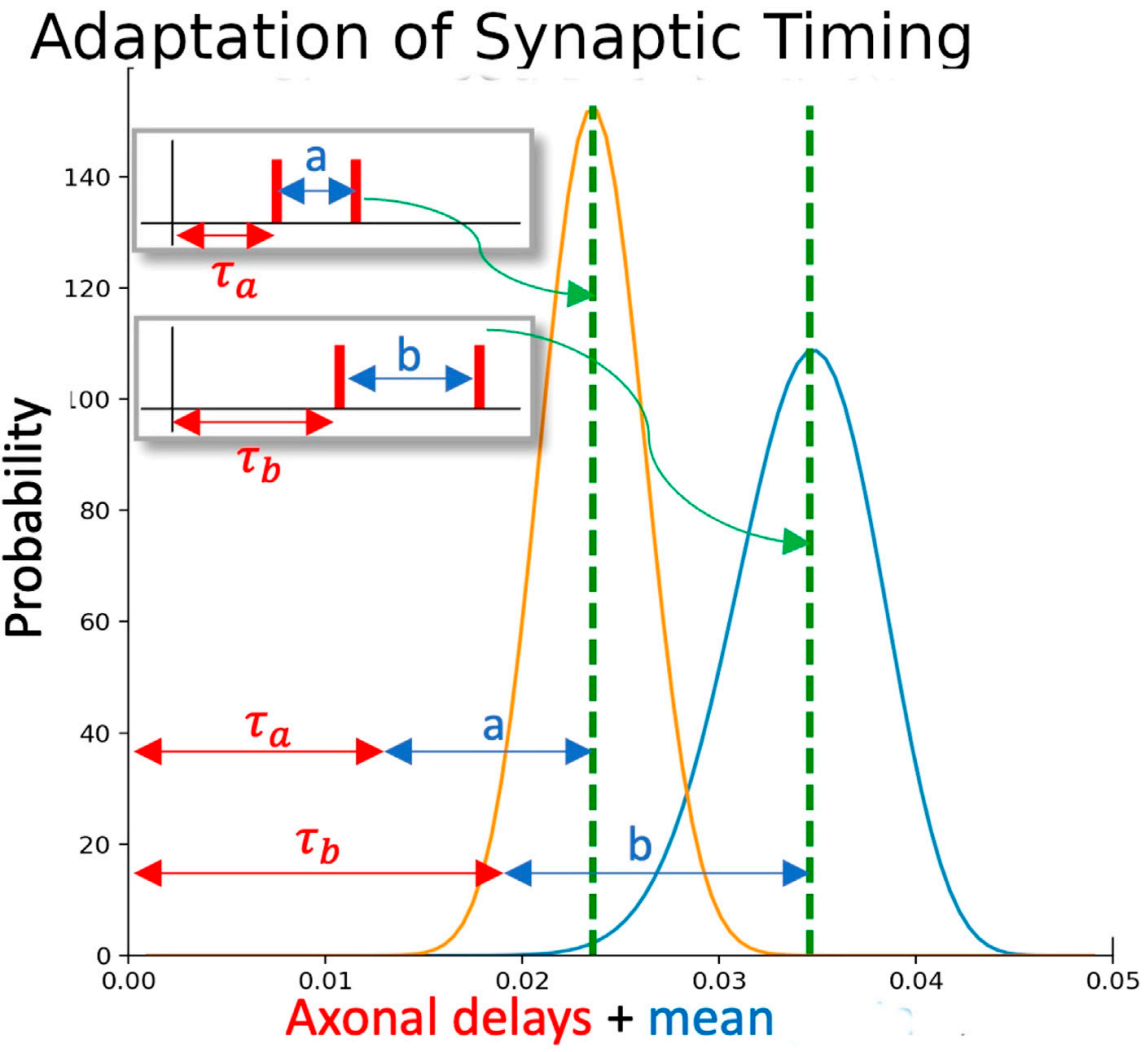
Adaptation of synaptic timing. Each ISI is associated with a particular distribution of sensitivities in the synapses. If one synapse (with yellow distribution) is activated by an ISI of a, and a second synapse (with blue distribution) is activated by an ISI of b, the overlap between the two distributions gives a window where the overall EPSP to the neuron will be raised. Both distributions are adapted if the postsynaptic neuron subsequently fires.

Now, we wish for functional synaptic clusters to learn to specialise on one or the other of a series of such timings within a spike train, so that different messages embedded within the signal may be independently decoded. Each synapse must thus be sensitive to a given delay (i.e., phase) within the some time window, and each neuron should have a series of input synapses whose timing sensitivities match the desired ISI (inter-spike interval) for one such timing, so that the neuron will fire with an output timing corresponding to the degree of match of the pattern. A very close match should produce a very early spike, an approximate match will produce a later spike, no match at all will prevent the neuron from spiking outright. To make the synapses sensitive to the delay, it will have an output transmission conditional on a delay distribution, where the peak of the distribution occurs at the exact match of the input spike to the tuned delay. Somewhat later and/or earlier input spikes will cause a lower current injection and hence retard the output time relative to a close match; a delay far away altogether will result in zero current injection. This mechanism implements the synapse that adapts itself to match the phase noise distribution N(ϕ) of subsection 2.1.1. One may think of the first spike of an ISI pair as priming the neuron to fire when a well-timed second spike arrives, and the second spike as triggering the output. This could be considered a limit case of burst gating (either excitatory (Borden et al., 2022) or inhibitory (Elson et al., 2002), where instead of a spike triggering transmission in a burst window, only a single spike with tuned timing is needed to trigger a comparable transmission. However, where in previous works, subsequent learning (or plasticity) only affected the strength of the synapse, here it affects the timing sensitivity of the synapse. We can see (Figure 2) that the overlap between the two distributions leads to an output firing probability distribution proportionate to the degree of timing match. Using this method, we will investigate spike trains with internal time structure, and attempt to decode separate data within such spike trains.

### 2.2 Methodology

In this paper, we explore the computational properties of resonant synapses when they are combined into functional synaptic clusters that are capable of decoding sequences of inter-spike intervals. In particular, we seek to demonstrate that these clusters have the following computational properties:

• The ability to ‘decode’ ordered sequences of inter-spike intervals (ISIs) across multiple time-structured spike sequences

• Enable the recognition of sequences of ISIs across multiple input units.

• Facilitate the decoding of such sequences in multiplexed neural encoding scenarios.

In this work we adopt the term ‘Interspike Interval’ (ISI) to refer not just to 2 immediately consecutive spikes, but to any arbitrary pairing of presynaptic spikes in some temporal order. Thus 2 different ISIs could ‘interleave’ such that one ISI had an intervening spike between the first spike of its pair and the second.

**FIGURE 3:**
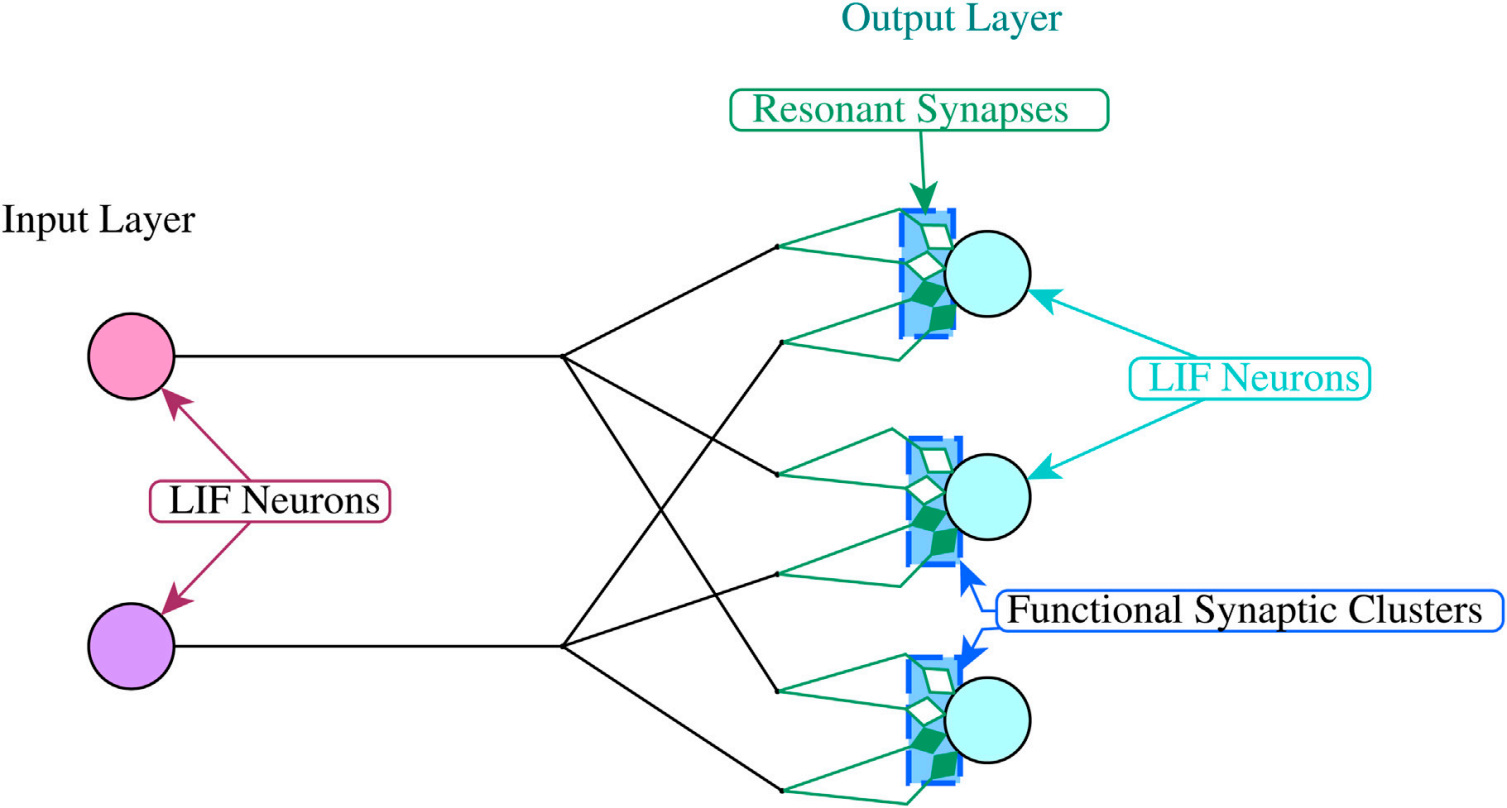
The network architecture.

We adopt a simple two layer spiking neural network architecture to demonstrate the above properties. Each network has an input layer and an output layer that are fully connected, with multiple synaptic connections between each input and each output neuron (See Figure 3). In our experiments we use the Leakey Integrate and Fire model for all neurons Gerstner and Kistler (2002).

$$\tau_{m}\frac{dV}{dt}=V_{r}-V+\frac{\tau_{m}\left(I_{\mathrm{syn}}+I_{os}\right)}{C_{m}};\;\text{ If }\left(V\geq V_{t}\right),V=V_{s}$$ (7)

The spike sequences from the input layer are generated using above threshold current injections to each of the input neurons at the time specified by the associated input patterns. The resonant synapses that are connected to each output neuron are grouped into functional synaptic clusters (Figure 3), where each cluster decodes the sequence of incoming interspike intervals for one input pattern.

The resonance property of our synaptic model is represented using a normal distribution whose parameters are adapted from the statistics of the in-coming interspike intervals. In particular, the mean of the distribution represents the mean interspike interval that the synapse will selectively respond to and is used to determine the transmission probabilities of the synapse (Figure 2):

$$P\left(T\left(s_{k},t_{i}\right)|t_{j},\mu_{k},\sigma_{k}\right)∼N_{\mu_{k},\sigma_{k}}\left(t_{i}-t_{j}\right)$$ (8)

where $T\left(s_{k},t_{i}\right)$ denotes a transmission event from synapse $s_{k}$ at time $t_{i}$ , and $t_{i}$ and $t_{j}$ are the arrival times in the synapse $s_{k}$ of any two spikes in a sequence where $t_{j}<t_{i}$ . $\mu_{k}$ and $\sigma_{k}^{2}$ are the mean and variance of the incoming ISIs statistics for synapse $s_{k}$ . In our model an ‘interspike interval’ is regarded as the time between any two spikes arriving at a synapse, regardless of whether are any intermediate spikes between $t_{i}$ and $t_{j}$ (such intermediate spikes are not ‘discarded’ and will continue to affect the dynamics of the synapse, but the term ISI is used here to refer to any identifiable spike pair, even if there are intervening spikes). This property is necessary for the model to be able to decode multiplexed spike trains.

When calculating the probability of a transmitting event in a given synapse $s_{k}$ , our resonant synapse model sums the probabilities of transmission from the interspike intervals arriving in $s_{k}$ between the last spike $t_{i}$ and all preceding spikes $t_{j}<t_{i}$ in the current phase cycle. To estimate the overall probability that the synapse will transmit after receiving incoming spike at $t_{i}$ , given all preceding spikes $t_{j}<t_{i}$ , we use the CDF of Equation 8 for synapse $s_{k}$ to calculate the area under the normal curve of a fixed-width interval from −a to a either side of each ISI. That is, between $N_{\mu_{k},\sigma_{k}}\left(\left(t_{i}-t_{j}\right)-a\right)$ and $N_{\mu_{k},\sigma_{k}}\left(\left(t_{i}-t_{j}\right)+a\right)$ summing over all interspike intervals in the current phase as follows:

$$\begin{array}{cc} P\left(T\left(s_{k},t_{i}\right)|\mu_{k},\sigma_{k},a\right) & =\underset{t_{j}<t_{i}}{\sum }\left(\Phi \left(\left(t_{i}-t_{j}\right)+a;\mu_{k},\sigma_{k},a\right)-\Phi \left(\left(t_{i}-t_{j}\right)-a;\mu_{k},\sigma_{k},a\right)\right)\\ & =\underset{t_{j}<t_{i}}{\sum }\left(\int_{-\infty }^{t_{i}+a}N_{\mu_{k},\sigma_{k}}\left(t\right)dt-\int_{-\infty }^{t_{i}-a}N_{\mu_{k},\sigma_{k}}\left(t\right)dt\right) \end{array}$$ (9)

Transmission events are generated using Bernoulli sampling based on Equation 9:

$$T\left(s_{k},t_{i}\right)=\left\{\begin{array}{cc} w\; & \text{if }r<P\left(T\left(s_{k},t_{i}\right)|\mu_{k},\sigma_{k},a\right)\\ 0\; & \text{otherwise} \end{array}\right.$$ (10)

where w is a fixed transmission weight for all synapses and r∼Uniform(0,1) .

Each synapse also has an independent time delay, which, as we shall see below, is critical in enabling clusters of such synapses to decode incoming spike trains. The functional synaptic clusters in this context have the role of coordinating the transmission times of active synapses so that specific sequences of inter-spike intervals result in an above threshold voltage of the post-synaptic neuron at the appointed time $t_{T}$ .

**FIGURE 4:**
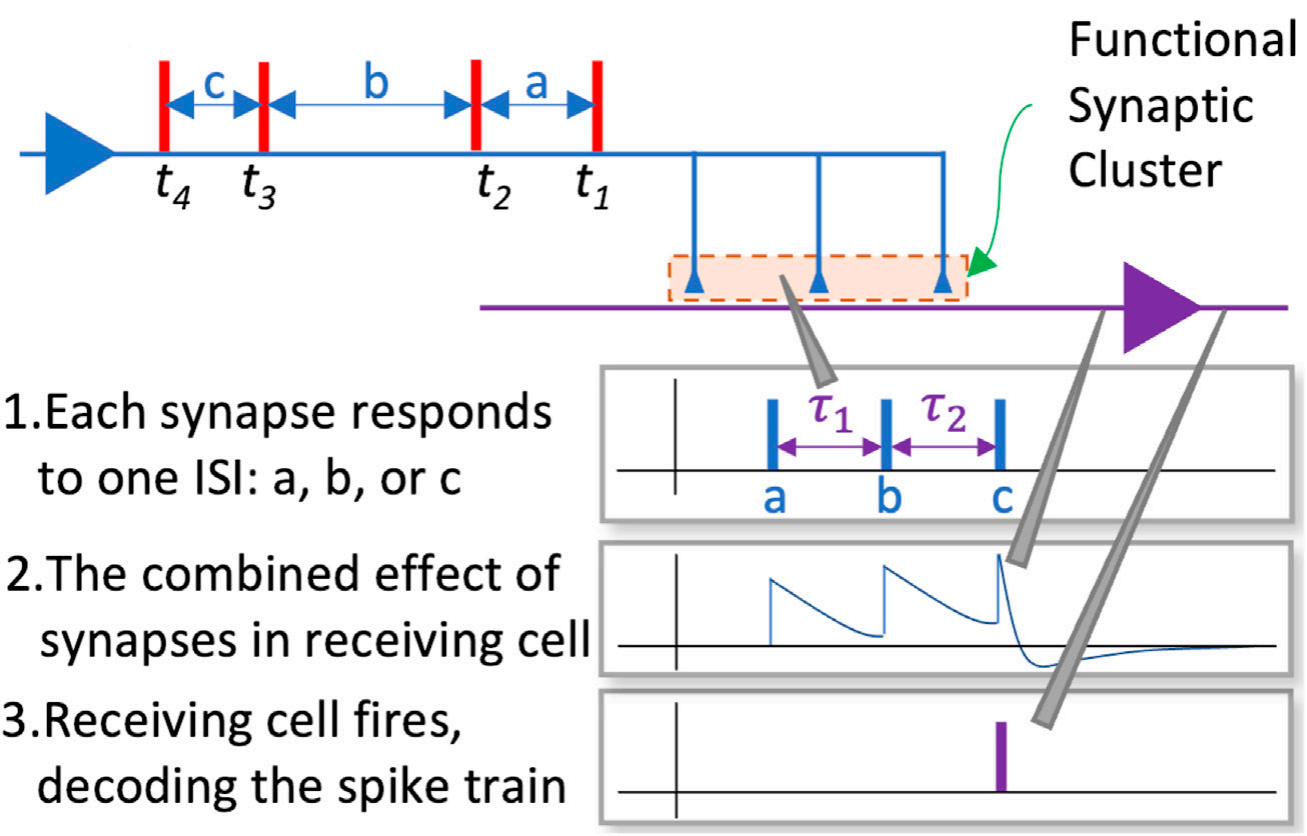
The allocation of ISIs to synapses.

The learning or adaptation in this model takes place in two phases. In the first phase each cluster assigns the incoming ISIs in the training sequence to a distinct synapse, enabling it to specialise on the statistics of that ISIs within that sequence over multiple presentations of the spike train. In the second phase the delays of each synapse in the cluster are adapted to facilitate the decoding of the sequence of ISIs it is being trained on. In the experiments reported below, the delays are initially randomised using samples from a uniform distribution, and the allocation of ISIs to resonant synapses was done using the temporal order of the incoming ISIs (see Figure 4). For example, if a cluster consisting of three synapses ( $s_{1}$ , $s_{2}$ , $s_{3}$ ) each with a randomised delay ( $d_{1}$ , $d_{2}$ , $d_{3}$ ) receives a spike train consisting of four spikes ( $t_{1}$ , $t_{2}$ , $t_{3},t_{4}$ ), and the delays are such that $d_{1}<d_{2}<d_{3}$ , then the cluster will allocate the first ISI $\left(t_{2}-t_{1}\right)$ to $s_{1}$ , the second $\left(t_{3}-t_{2}\right)$ to $s_{2}$ and the third $\left(t_{4}-t_{3}\right)$ to $s_{3}$ .

The statistics of the ISIs are processed using Welford’s online algorithm which facilitates the calculation of the variance in one pass (Chan et al., 1983). The formulae for the online algorithm are shown in Equation 11.

$$\begin{array}{cc} {\bar{x}}_{n} & ={\bar{x}}_{n-1}+\frac{x_{n}-{\bar{x}}_{n-1}}{n}\\ M_{2,n} & =M_{2,n-1}+\left(x_{n}-{\bar{x}}_{n-1}\right)\left(x_{n}-{\bar{x}}_{n}\right)\\ \sigma_{n}^{2} & =\frac{M_{2,n}}{n}\\ s_{n}^{2} & =\frac{M_{2,n}}{n-1} \end{array}$$ (11)

where $x_{n}$ is the new sample which, in our case, are interspike intervals $t_{i}-t_{j}$ , ${\bar{x}}_{n}$ is the mean of the first n samples, $\sigma_{n}^{2}$ is the biased sample variance, and $s_{n}^{2}$ is the unbiased sample variance. $M_{2,n}$ holds the sum of square differences of the mean. Updates to the mean and variance of each synapse are only permitted if the order of the incoming ISI matches the allocation made by the cluster to that synapse.

The adaptation of the synaptic delays which takes place in the second phase of the training enables the cluster to successfully decode the incoming sequence of ISIs. This is achieved by aligning the delays such that each transmission meets the following criteria:

$$t_{\tau }=\left\{\begin{array}{cc} -\tau_{m}\log_{e}\left(\left(v_{\tau }-R_{m}I_{s}\right)/v_{b}\right)\; & v\neq v_{r}\\ 0\; & v=v_{r} \end{array}\right.$$ (12)

where $t_{\tau }$ is the target time delay between successive transmissions, $\tau_{m}$ is the decay constant and $v_{r}$ the resting potential in Equation 7, $v_{\tau }$ is the target potential after t time steps ( $v_{\tau }$ will either be $v_{b}$ for a below post-synaptic threshold transmission which is not the last in the decoding sequence, or $v_{a}$ for an above threshold transmission for the last in the decoding sequence), and $R_{m}=\frac{\tau_{m}}{C_{m}}$ from Equation 7. When $v=v_{r}$ it may be assumed that the spike is the first in the sequence since there has been enough time for the neuron to return to resting potential. This equation is obtained by solving the closed form LIF update formula shown in Equation 13.

$$V_{\tau }=R_{m}I_{s}+V_{b}e^{-\frac{t}{\tau_{m}}}$$ (13)

for t and setting $t_{\tau }$ to the value that will yield $V_{\tau }$ . The incremental updates to the delays of resonant synapses are made according to the following:

$$d_{k}=d_{k}+w_{d}\left(t_{\tau }-t\right)$$ (14)

where $w_{d}$ is the delay update weight, $t_{\tau }$ is the target transmission time for that synapse, and t is the current transmission time.

Our model determines the transmission time of each synapse in a cluster using the ordering of the ISIs that were allocated to each synapse in the first phase of training. To illustrate this process we return to the example of the three synapses given previously [ $s_{1}$ , $s_{2}$ , $s_{3}$ ] which were allocated the following ISIs: $s_{1}\text{ allocated }t_{2}-t_{1}$ , $s_{2}\text{ allocated }t_{3}-t_{2}$ and $s_{3}\text{ allocated }t_{4}-t_{3}$ . Since $s_{3}$ was allocated the last ISI in this sequence, it will be responsible for securing an above threshold response from the post-synaptic neuron when the fourth spike of the sequence $t_{4}$ is received. To achieve this, synapse $s_{3}$ will use Equation 12 (with $v_{\tau }=v_{a}$ ) to incrementally adjust its delay $d_{3}$ over several iterative presentation of the four-spike pattern so that the arrival of the fourth spike will ultimately coincide with the timing of the desired above threshold response in the post-synaptic neuron (i.e., $t_{T}$ ).

Synapse $s_{2}$ , which responds to the second ISI in this sequence, will need to deliver a below threshold transmission using Equation 12 (with $v_{\tau }=v_{b}$ ) which needs to be timed so that the post-synaptic voltage will reaching its above resting value of $v_{b}$ just as the third spike arrives in the synapse. Once again, it achieves this using Equation 12 to interactively adjust its time delay accordingly. Similarly, synapse $s_{1}$ adjusts its time delay so that it can deliver a below threshold transmission $\left(v_{\tau }=v_{b}\right)$ just as the second pre-synaptic spike arrives. In this way, only a spike train consisting of three ISIs that match the pattern ( $t_{1}$ , $t_{2}$ , $t_{3},t_{4}$ ) will result in a spike at $t_{T}$ in the post-synaptic neuron signalling that this spike train has been successfully recognised or decoded.

## 3 Results

The objectives of the experimental work presented are to demonstrate that resonant synapses combined into functional synaptic cluster can:

1. Decode sequences of ISIs from separate input sources
2. Decode sequences of ISIs that are distributed across multiple input sources
3. Decode multiplexed ISI sequence signals

The experimental work presented in this section involved the use of the Brian2 simulator^1^ to create networks of LIF neurons (Equation 7) arranged in two connected layers, with layer 1 generating the input signals and layer 2 decoding those signals and generating the output signals (Figure 3)^2^.

The connections between the input and the output neurons were modelled using resonant synapses as described in the previous section. Each input neuron had multiple time-delayed resonant synapse connections with each of the output neuron. Each output neuron had one functional synaptic cluster which included all the resonant synapses that were connected to it from the input layer. In this way, the output units could decode sequences of ISIs that were distributed across multiple input units.

Each sequence of input spikes and the corresponding decoding output spikes occur within a cyclic 100 m window or phase that is synchronous for all input and output units. The target decode time was fixed in the range $50\text{ms}<t_{T}<100\text{ms}$ for each experimental run. It is important to note that the phase duration and the target decode time together put a limit on the number of sequential ISIs that the network can decode. This is because the delays between the transmissions of each synapse (which is responsible for detecting one of the ISIs) have to line up sequentially to successfully decode the sequence and generate a post-synaptic spike at $t_{T}$ . Since the synaptic delays are determined by Equation 12, it is the constants used in that equation such as $\tau_{m}\text{ and }v_{r}$ that determine the time between successive transformations, and consequently the maximum number of ISIs that can be decoded at $t=t_{T}$ . In the experiments we present below, the maximum number of ISIs that could reliably be decoded (given that all input spike times were generated randomly within set bounds) is three (i.e. 4 sequential spikes).

The performance of the networks was evaluated using the previously discussed distance measure shown in Equation 4; (Lyttle and Fellous, 2011), comparing the sequence of spikes emitted by each output neuron with the target spike output for the corresponding input pattern. Each output neuron is induced to emit a spike to mark the start of the 100 m phase window. The target spike sequence for an output neuron that is designated to decode the current input pattern therefore consists of two spikes: one at t=0 and the other at the target decode time $t_{T}$ . The target spike sequence for an output neuron that is not designated to decode the current input pattern consists of just one spike at t=0 . Target and actual spike sequences are convolved with a Gaussian kernel and then compared using Equation 4. The resulting distance measures are reported in the tables below.

The model’s constants shown in Equation 7 through to 12 are summarised in Table 1.

**TABLE 1:** Initial parameters.

Constant	Equation(s)	Value	Condition
$\tau_{m}$	Equation 7	10	-
$C_{m}$	Equation 7	1.0	-
$V_{t}$	Equation 7	1.0	-
a	Equation 8	2 m	-
w	Equation 10	0.8	multiple ISI sequence
w	Equation 10	1.8	single ISI
$v_{r}$	Equation 12	$v_{a}=1.5$	Above threshold transmission
$v_{r}$	Equation 12	$v_{a}=0.9$	Below threshold transmission
$v_{s}$	Equation 12	0	First spike
$v_{s}$	Equation 12	$v_{a}$	Not first spike
$w_{d}$	Equation 14	0.4	-

Model variables were randomised at the start of each experimental run by making draws from a uniform distribution. The maximum and minimum values of that distribution are shown in Table 2.

**TABLE 2:** Maximum and minimum range of initial values of randomised variables.

Variable	Minimum value	Maximum value
synaptic delay	5 m	20 m
resonance mean (μ)	5 m	20 m
resonance standard deviation (σ)	2 m	5 m

The remainder of this section presents three sets of experimental results corresponding to the three objectives listed above. Each set consists of multiple experiments with different configurations of input and output neurons, and different input patterns. The times of the spikes in the input patterns are randomly generated using a ‘stick breaking’ process Sethuraman (1994). The models used in all of the following experiment had 4 resonant synapses from each input to every output neuron. All of the synapses that impinged on the same output neuron were recruited into the same functional synaptic cluster.

**FIGURE 5:**
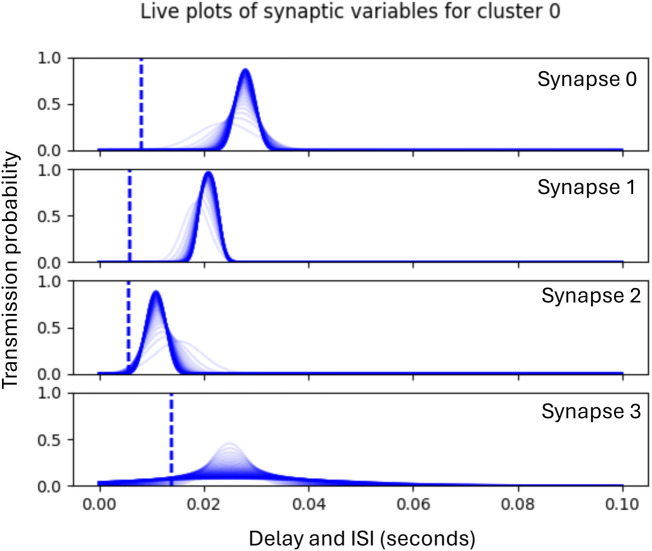
Graphs showing historic plots from initial values (faint blue) to final values (dark blue) of the adaptation of synaptic transmission probabilities during Phase 1. Note that these plots are not probability distributions, but show the probability of transmission sampled across the range of ISIs shown.

Each experimental run consisted of 300 epochs, where the duration of an epoch is equal to the phase duration (100 m) during which a ‘jittered’ version of the input pattern is presented to the input layer, and a response from the output layer is generated. No learning or adaptation is applied in first and the last 50 epochs, enabling the collection of pre-training and post-training performance metrics. Phase 1 of learning begins from epoch 51, during which time the clusters allocate incoming ISIs to the synapses which in turn learn the statistics of the ISIs assigned to them. The graph in Figure 5 shows an example illustrated with historic plots of the probability of transmission for each of four synapses within the same functional cluster during Phase 1 adaptation. In this case, synapse 2 has adapted to the statistics of the first incoming ISI, synapse 1 to the second, and synapse 0 to the first. Synapse 3 has not been allocated an ISI and so its standard deviation is progressively increased to prevent unwanted transmissions. The dotted line shows the initial randomised delay, which is adapted during phase 2 of learning.

**FIGURE 6:**
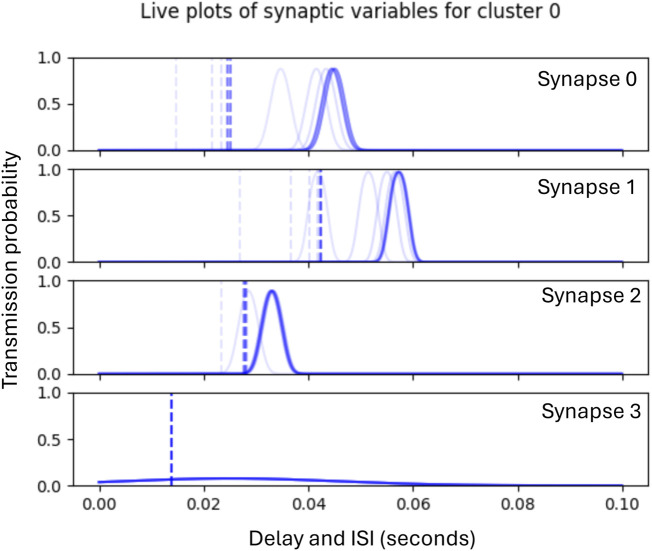
Graphs showing historic plots (faint to dark blue) of the adaptation of synaptic delays during Phase 2

Phase 1 lasts for 180 epochs, after which Phase 2 begins the adaptation of the synaptic delays which continues for a further 20 epochs. Figure 6 illustrates the subsequent Phase 2 adaptation of the delays of the synapses shown in Figure 5.

Each of the results shown in the tables below are averaged over 10 experimental runs, each starting with one or more randomised input patterns for the network to learn.

.

### 3.1 Experiment 1: separate input sources

This first suite of experiments seeks to establish that resonant synapses that are organised into functional synaptic clusters can learn to successfully decode incoming sequences of ISIs. We are interested in how this model performs as the complexity of this decoding task increases both in terms of the number of ISIs making up the input sequence, and in terms of the complexity of the network (i.e., the number of input and output units), and where sequences of ISIs to be decoded were presented on separate input units.

Table 3 shows the mean and standard deviation of the distance measure using Equation 5 for three sets of experiments. Columns 2 and 3 show the number of input and output units in each experimental subset, and columns 5 to 10 show the mean and standard deviation of the distance measure with respect to the target output both prior and post training, with the number of ISIs in the input sequence increasing from 1 to 3. Each row of the table is showing means and standard deviations that have been averaged across 10 randomised network parameter sets (Table 2) and 10 randomised spike trains that were jittered prior to presentation a the input layer.

**TABLE 3:** Decoding sequences of ISIs from separate input sources. The table reports the mean and standard deviation of the distance measure shown in Equation 5 averaged across the experimental runs.

				1 ISI		2 ISIs		3 ISIs	
Exp	No.	No.	Stats	Pre-train	Post-train	Pre-train	Post-train	Pre-train	Post-train
No.	Inputs	Outputs							
1.1	1	1	Mean	0.19863	0.01649	0.21073	0.09346	0.29789	0.15690
			St.Dev	0.06003	0.00706	0.04651	0.03999	0.06388	0.07941
1.2	1	2	Mean	0.12898	0.03306	0.18494	0.07365	0.22596	0.14233
			St.Dev	0.01766	0.02830	0.03514	0.04343	0.06412	0.07429
1.3	2	4	Mean	0.09132	0.04610	0.11934	0.05146	0.18573	0.07962
			St.Dev	0.06003	0.00706	0.04651	0.03999	0.06388	0.07941

The post-learning results show a clear improvement in performance over the pre-training outcomes. It is also clear that as the model and the data complexity increase, the averaged distance measures also increase as expected. It worth commenting, however, on the fact that the distance measure on the pre-trained networks already have a low similarity score. The primary reason for this is that the randomisation of the means, standard deviations and time delays for the resonant synapses at the start of each experimental run must be done within the constraints of the phase duration (100 m). These randomisations have a tendency to produce initial parameters that are often not far from the optimal ones for decoding. Nevertheless, the results in Table 3 demonstrate that learning has taken place. Using Equation 5, we see, for example, that the decoding probability has increased in Experiment 1.1 for 2 ISIs from 0.79 (79%) to 0.91 (91%).

### 3.2 Experiment 2: decode sequences of ISIs that are distributed across multiple input sources

The second suite of experiments explore the capacity of this model to decode time structured spike trains where the ISIs that make up the sequence are distributed across multiple inputs. As before, the columns of Table 4 show the network structure and the number of ISIs presented to each of the input units. Given the limit of 3-4 ISIs per pattern to be learned, this model was unable to process 3 ISIs per input unit in experiment 2.1, 2.2, and 2.3, or 2 ISIs per input unit in experiment 2.3. The remaining results demonstrate that learning has taken place between pre-training and post training phases. Once again from Equation 5, in Experiment 2.1 for 2 ISIs, the decode probability increases from 0.65 (65%) to 0.82 (82%). Similar probabilities can be derived for all the experiments conducted.

**TABLE 4:** Decoding sequences of ISIs that are distributed across multiple input sources.

				1 ISI		2 ISIs		3 ISIs	
Exp	No.	No.	Stats	Pre-train	Post-train	Pre-train	Post-train	Pre-train	Post-train
No.	Inputs	Outputs							
2.1	2	1	Mean	0.21239	0.10239	0.35427	0.17567	-	-
			St.Dev	0.04158	0.03765	0.05623	0.09353	-	-
2.2	2	2	Mean	0.13026	0.08799	0.28212	0.13777	-	-
			St.Dev	0.01671	0.01844	0.04634	0.04811	-	-
2.3	4	3	Mean	0.16728	0.08072	-	-	-	-
			St.Dev	0.04328	0.01483	-	-	-	-

### 3.3 Experiment 3: multiplexed input signals

This third suite of experiments explores the capacity of this model for decoding multiple distinct sequences of ISIs that have been combined together into one input. The training sequence for these experiments differs from that used in the previous experiments; in this case a number of random patterns (sequences of ISIs) are generated and the model is trained on jittered versions of them as before. However, in the test phase, these spike patterns are merged into one sequence. As before, the spikes in this combined sequence are jittered and the model is evaluated on its performance in decoding them both before and after training. The results are summarised in Table 5.

**TABLE 5:** Decoding multiplexed sequences of ISIs.

				1 ISI		2 ISIs		3 ISIs	
Exp	No.	No.	Stats	Pre-train	Post-train	Pre-train	Post-train	Pre-train	Post-train
No.	Inputs	Outputs							
3.1	1	2	Mean	0.34180	0.15499	0.55112	0.25910	0.67704	0.41644
			St.Dev	0.06404	0.07522	0.05525	0.07972	0.04856	0.06802
3.2	1	3	Mean	0.56882	0.24305	0.73541	0.36983	0.81643	0.48556
			St.Dev	0.07949	0.03310	0.05066	0.04408	0.03036	0.02547

Unsurprisingly, the results show that the multiplexed input signals results in a significant increase in the distance measures as the complexity of the signals, the number of ISIs and the number of merged patterns are increased. Despite this, the model is able to improve on the distance measure scores after training, suggesting that it may have the capacity to decode multiplex time-structured spike trains.

There are some clear limitations to the experimental approach we have taken here. One limiting factor is that all models are trained with a fixed number of epochs for both phases of adaptation. It may be that longer training regimes might have resulted in better performance overall. It was our intention, however, to understand how model and pattern complexity affected the decoding challenge, and so consistent training times has helped to shed some light on this, whilst also demonstrating that functional clusters of resonant synapses have the potential to decode time structured spike trains under various conditions.

## 4 Discussion

The work we have done thus far develops the basic mechanism for learning and subsequently decoding time-structured spike trains - i.e., ones where information is coded in the relative phase of spikes. As has been seen in biology (Kayser et al., 2009), this potentially permits both higher information content and greater robustness to noise. Furthermore, by developing formal models for spike-train similarity and decoding, we are able to translate measures of spike similarity directly into decode probabilities, providing a constructive route to design of functional neural networks based on temporal coding, with known probability of information retrieval given a desired information density. These models could additionally be used to develop network-level as opposed to synapse-level learning rules, including supervised learning algorithms such as backpropagation (without the traditionally associated difficulties concerning the non-differentiability of a spike signal). Future work will build larger networks and libraries of such learning rules, allowing functional neural networks that can be integrated into real-time, real-world systems.

Further exploration of the information capacity of a temporally-coded neural network remains an interesting problem. Although Kayser et al. (2009) indicates there are gains to be had, Ikeda and Manton (2009) show that the phase difference between 2 distinct spike codes cannot be arbitrarily small, at least not if the decode is to be reliable. However, in real-world problems, completely reliable decoding may not be necessary. Some ‘error’ in computed output may be tolerable if results are not needed to arbitrary precision, e.g., when computing motor commands for a given set of outputs on a robot (where, in any case, the motors themselves have finite command tolerances). Furthermore, if spike-codes are chosen so that ‘similar’ phase codes (i.e., ones that lie adjacent to each other in the word space of Equation 2), then errors are bounded to similar semantic meanings, in the same way as is achieved in conventional neural networks through suitable choice of embeddings. Further formal work is clearly required to characterise the information-processing capabilities of temporal spiking neural networks under various bounds on intrinsic noise and symbol error. A major gap still exists likewise in formal convergence proofs and learning models for large-scale temporal spiking networks; the work presented here provides some foundations, but the field as a whole still lags behind the maturity achieved in conventional neural networks.

Although resonant synapses (RSs) (Crook et al., 2023) were initially inspired by resonant neuronal groups (RNGs) (Aoun, 2022) and we find similarities between both approaches (e.g., ISI processing), RNGs are neuron-based while RSs are dendritic. In addition, RS employs probability distributions and statistics in decoding inputs while, RNGs follow nonlinear dynamical systems methodology. We are exploring methods to combine both a dynamical systems approach and the probabilistic approach to develop stochastic dynamical neural networks not dependent on either noiseless signalling nor on discrete-time processing models.

The recent work of Agnes and Vogels (2024) on codependent inhibitory and excitatory plasticity explores how the interplay between excitatory and inhibitory synaptic changes maintains network stability while enabling effective learning. By incorporating these principles, the proposed temporal synaptic learning model could achieve better balance in neural activity, reduce the risk of runaway excitation or inhibition, and enhance its ability to process complex, multiplexed spike patterns in a biologically plausible manner. It provides insights into how balanced synaptic modifications can enhance network stability and learning efficiency. Incorporating such mechanisms could improve the robustness and adaptability of the proposed temporal synaptic learning model.

The ability to decode different spike patterns implies an orthogonality of inputs. Indeed, there is some biological evidence to suggest this: rats use their whiskers to encode the three-dimensional location of objects through an orthogonal, triple-code scheme (Knutsen and Ahissar, 2009). In this model, vertical coordinates (elevation) are encoded spatially by the identity of activated sensory neurons, horizontal coordinates (azimuth) are encoded temporally by the timing of activation, and radial coordinates (distance) are encoded by the intensity of activation. These orthogonal inputs are mutually independent, allowing individual primary afferents to encode all three dimensions during a single whisker-object contact simultaneously. Such a coding scheme reduces ambiguity and simplifies decoding circuits, enabling efficient tactile perception during active sensing. Multidimensional sensory and processing systems such as this could likewise be employed, e.g., in mobile robotics, enabling efficient integration of multimodal sensory input within constrained wiring and power budgets (Yu et al., 2023).

## 5 Conclusion

We have demonstrated a mechanism for temporal synaptic learning able to discriminate different patterns of phase-coded spiking signals. This enables a new class of neural network able to operate and learn in real time, with capabilities much closer to the known abilities of biological systems, in contrast to typical modern large-scale neural networks trained offline and requiring massive datasets, with representations distributed over a large number of connections. With this new learning and coding model, multiple classes of information can be transmitted and computed upon simultaneously, by the same circuitry, at potentially significant power savings. Even what is to be computed does not need to be predecided or wired in, in advance: such networks, which have already learnt to compute certain results can reuse data latent in the signals being sent to potentially learn new classes of result that had hitherto been unimportant or insignificant. Discarding the fundamentally discrete-time, synchronous processing model of both conventional neural networks and of digital circuitry has always been a desideratum with real-world systems; we have introduced a solution to the problem of learning in such continuous-time processing that makes it feasible.

## Data Availability

The original contributions presented in the study are included in the article, further inquiries can be directed to the corresponding author.

## Footnotes

1
https://briansimulator.org

2
https://github.com/ntcrook/NeuromorphicCode
